# Supplementary material for: Negative regulation of ABA signaling by WRKY33 is critical for Arabidopsis immunity towards Botrytis cinerea 2100
Source: eLife. 2015 Jun 15;4:e07295. doi: 10.7554/eLife.07295 (PMC4487144; doi:10.7554/eLife.07295)
Supplement: Supplementary file 1. — Summary of identified WRKY33 binding sites 14 hr post B. cinerea 2100 inoculation. DOI: http://dx.doi.org/10.7554/eLife.07295.023 [file elife07295s001.docx]

**Supplementary file 1** Summary of identified WRKY33 binding sites within the Arabidopsis genome 14 h post *B. cinerea* 2100 infection.

Note:

^1)^ A peak region in a replicate is counted as reproducible, if it overlaps with a peak region in the other replicate by at least 50% of the length of the smaller peak region.

^2)^ A peak region is counted as high confidence region (within a sample), when it is identified against all (two/three) negative controls used for peak calling.

^3)^ A peak region in the pooled sample is counted as consistent, if it overlaps with a reproducible peak region in both of the original replicates by at least 50% of the length of the smaller region.

- For both replicates, peak calling was performed against two different negative controls: a mock-treated ChIP control (mock_IP) and a Bc-treated input control. Bc_input samples were identically processed as the IP samples except that no HA-antibody was added in the ChIP assay.

- For the WT-type negative controls (WT_IP_Bc_vs_mock), no peaks were observed, therefore only one replicate (replicate 2) was performed and analyzed here.
