# Supplementary material for: Negative regulation of ABA signaling by WRKY33 is critical for Arabidopsis immunity towards Botrytis cinerea 2100
Source: eLife. 2015 Jun 15;4:e07295. doi: 10.7554/eLife.07295 (PMC4487144; doi:10.7554/eLife.07295)
Supplement: Supplementary file 2. — List of confirmed WRKY33 target genes. DOI: http://dx.doi.org/10.7554/eLife.07295.024 [file elife07295s002.docx]

**Supplementary file 2** List of confirmed WRKY33 regulated target genes.

| Gene | Description | Test methods | |
| --- | --- | --- | --- |
| AT2G30750 | CYP71A12 | ChIP-qPCR |  |
| AT2G30770 | CYP71A13 | ChIP-qPCR | LinDA-qPCR |
| AT3G26830 | PAD3 | ChIP-qPCR | LinDA-qPCR |
| AT5G05730 | AMT1 | ChIP-qPCR |  |
| AT5G22570 | WRKY38 | ChIP-qPCR |  |
| AT5G26170 | WRKY50 | ChIP-qPCR |  |
| AT4G23810 | WRKY53 | ChIP-qPCR |  |
| AT4G11070 | WRKY41 | ChIP-qPCR |  |
| AT5G49520 | WRKY48 | ChIP-qPCR |  |
| AT2G40740 | WRKY55 | ChIP-qPCR |  |
| AT3G44350 | NAC061 | ChIP-qPCR | LinDA-qPCR |
| AT5G22380 | NAC090 | ChIP-qPCR |  |
| AT2G26650 | AKT1 | ChIP-qPCR | LinDA-qPCR |
| AT3G14440 | NCED3 | ChIP-qPCR | LinDA-qPCR |
| AT1G30100 | NCED5 | ChIP-qPCR | LinDA-qPCR |
| AT5G45340 | CYP707A3 | ChIP-qPCR |  |
| AT1G16090 | WAKL7 |  | LinDA-qPCR |
| AT5G44280 | RING1A |  | LinDA-qPCR |
| AT5G45110 | NPR3 |  | LinDA-qPCR |
| AT5G20960 | AAO1 |  | LinDA-qPCR |
| AT1G01480 | ACS2 |  | LinDA-qPCR |
| AT3G55970 | JRG21 |  | LinDA-qPCR |
| AT5G47230 | ERF5 |  | LinDA-qPCR |
| AT3G23240 | ERF1 |  | LinDA-qPCR |
| AT4G11280 | ACS6 | ChIP-qPCR |  |
| AT1G72520 | LOX4 | ChIP-qPCR |  |

Note: For ChIP-qPCR, the ChIP DNA was used as template. The ChIP-DNA after the linear DNA amplification step (LinDA-DNA) was used as template for LinDA-qPCR.
