# Supplementary material for: Negative regulation of ABA signaling by WRKY33 is critical for Arabidopsis immunity towards Botrytis cinerea 2100
Source: eLife. 2015 Jun 15;4:e07295. doi: 10.7554/eLife.07295 (PMC4487144; doi:10.7554/eLife.07295)
Supplement: Supplementary file 3. — List of WRKY33 regulated target genes involved in cell death. DOI: http://dx.doi.org/10.7554/eLife.07295.025 [file elife07295s003.docx]

**Supplementary file 3** List of WRKY33 regulated target genes involved in cell death.

| Gene | Description | score_ChIP | log2FC Bc KO-WT |
| --- | --- | --- | --- |
| AT5G05730 | AMT1 | 22,94 | -1,60 |
| AT2G30770 | CYP71A13 | 12,74 | -3,17 |
| AT3G26830 | CYP71B15 | 56,29 | -1,61 |
| AT4G23810 | ATWRKY53 | 49,21 | 1,45 |
| AT5G22570 | ATWRKY38 | 13,52 | 4,50 |
| AT2G38470 | ATWRKY33 | 30,45 | -3,32 |
| AT5G60900 | RLK1 | 28,11 | 1,17 |
| AT2G32680 | AtRLP23 | 54,73 | 1,06 |
| AT4G35600 | CST | 17,28 | 1,64 |
| AT4G23170 | CRK9 | 18,13 | 1,33 |
| AT3G09830 | protein kinase | 39,86 | 1,16 |
| AT5G48380 | BIR1 | 29,67 | 1,88 |
| AT4G26070 | ATMEK1 | 26,55 | 1,36 |
| AT1G01560 | ATMPK11 | 14,73 | 2,61 |
| AT5G01540 | LECRKA4.1 | 24,64 | 1,07 |
| AT4G04490 | CRK36 | 22,37 | 2,01 |
| AT1G18890 | ATCDPK1 | 13,03 | 1,27 |
| AT3G45290 | ATMLO3 | 22,02 | 1,41 |
| AT1G11310 | ATMLO2 | 24,36 | 1,09 |
| AT2G39200 | ATMLO12 | 40,71 | 2,06 |
| AT5G61210 | ATSNAP33 | 39,65 | 1,39 |
| AT1G19250 | FMO1 | 11,40 | 1,75 |
| AT3G22160 | JAV1 | 22,09 | 1,16 |
| AT3G57260 | AtPR2 | 20,18 | 3,78 |
| AT4G14400 | ACD6 | 20,11 | 1,04 |
| AT1G29690 | CAD1 | 20,96 | 1,02 |
| AT1G07000 | ATEXO70B2 | 17,84 | 1,32 |
| AT3G11840 | PUB24 | 24,07 | 1,11 |
| AT1G76970 | Target of Myb1 | 16,21 | 1,63 |
| AT3G49350 | RabGAP/TBC domain | 11,75 | 1,20 |
| AT4G14365 | XBAT34 | 53,67 | 1,70 |
| AT3G01830 | CML40 | 17,98 | 2,09 |
| AT2G30550 | DALL3 | 14,30 | 1,44 |
| AT5G45110 | ATNPR3 | 11,61 | 1,78 |
| AT5G50200 | ATNRT3.1 | 14,59 | 1,87 |
| AT5G40780 | LHT1 | 22,52 | 1,38 |
| AT4G39670 | glycolipid transfer | 18,06 | 1,26 |
| AT5G44070 | ARA8 | 27,54 | 1,20 |
| AT5G24530 | DMR6 | 16,85 | 2,73 |
| AT1G08450 | AtCRT3 | 41,77 | 1,94 |
| AT4G34150 | calcium-dependent | 13,10 | 1,16 |
| AT4G12470 | AZI1 | 33,92 | 2,13 |
