# Supplementary material for: Negative regulation of ABA signaling by WRKY33 is critical for Arabidopsis immunity towards Botrytis cinerea 2100
Source: eLife. 2015 Jun 15;4:e07295. doi: 10.7554/eLife.07295 (PMC4487144; doi:10.7554/eLife.07295)
Supplement: Supplementary file 4. — List of WRKY33 regulated target genes associated in the GO category ‘kinase activity’. DOI: http://dx.doi.org/10.7554/eLife.07295.026 [file elife07295s004.docx]

**Supplementary file 4** List of WRKY33 regulated target genes associated with the GO term “kinase activity”.

| Gene | Description | score_ChIP | log2FC Bc KO-WT |
| --- | --- | --- | --- |
| AT5G48380 | BIR1 | 29,67 | 1,88 |
| AT4G08850 | LRR-RLP | 17,63 | 1,30 |
| AT2G25440 | RLP20 | 16,99 | 1,11 |
| AT2G32680 | RLP23 | 54,73 | 1,06 |
| AT3G11080 | RLP35 | 16,14 | 2,53 |
| AT3G28890 | RLP43 | 13,88 | 2,22 |
| AT1G47890 | RLP7 | 11,05 | 1,55 |
| AT1G51850 | LRR-PK | 15,51 | 2,91 |
| AT1G51790 | LRR-PK | 13,10 | 2,22 |
| AT3G14840 | LRR-PK | 28,68 | 1,26 |
| AT4G11480 | CRK32 | 15,58 | 1,76 |
| AT4G23170 | EP1 | 18,13 | 1,33 |
| AT4G23220 | CRK14 | 72,22 | 1,17 |
| AT1G70520 | CRK2 | 14,66 | 1,08 |
| AT4G23320 | CRK24 | 28,25 | 1,54 |
| AT4G04490 | CRK36 | 22,37 | 2,01 |
| AT4G23150 | CRK7 | 14,37 | 1,86 |
| AT2G32800 | LECRK-S.2 | 24,78 | 1,48 |
| AT4G04960 | LECRK-VII.1 | 40,93 | -1,75 |
| AT5G01540 | LECRK-VI.2 | 24,64 | 1,07 |
| AT5G01550 | LECRK-VI.3 | 26,55 | 2,57 |
| AT4G27300 | G-type | 19,12 | -1,00 |
| AT1G61460 | G-type | 12,39 | 1,15 |
| AT1G61420 | G-type | 24,14 | 1,12 |
| AT1G11330 | G-type | 17,06 | 1,25 |
| AT5G63650 | SNRK2.5 | 16,00 | -1,42 |
| AT5G47850 | CCR4 | 46,02 | 2,34 |
| AT4G35600 | CONNEXIN 32 | 17,28 | 1,64 |
| AT3G09830 | CCR-like | 39,86 | 1,16 |
| AT2G17220 | KIN3 | 18,83 | 1,02 |
| AT5G42440 | AT5G42440 | 25,14 | 1,34 |
| AT5G38210 | AT5G38210 | 24,92 | 1,06 |
| AT1G67000 | AT1G67000 | 16,36 | 1,12 |
| AT5G47070 | AT5G47070 | 18,48 | 1,00 |
| AT2G47060 | PTI1-4 | 23,08 | 1,13 |
| AT1G65790 | RK1 | 13,17 | 1,70 |
| AT5G60900 | RLK1 | 28,11 | 1,17 |
| AT1G01560 | MPK11 | 14,73 | 2,61 |
| AT4G26070 | MEK1 | 26,55 | 1,36 |
| AT1G18890 | CDPK1 | 13,03 | 1,27 |
| AT1G21270 | WAK2 | 35,90 | 1,01 |
