# Supplementary material for: Negative regulation of ABA signaling by WRKY33 is critical for Arabidopsis immunity towards Botrytis cinerea 2100
Source: eLife. 2015 Jun 15;4:e07295. doi: 10.7554/eLife.07295 (PMC4487144; doi:10.7554/eLife.07295)
Supplement: Supplementary file 5. — List of WRKY33 target genes encoding transcription factors. DOI: http://dx.doi.org/10.7554/eLife.07295.027 [file elife07295s005.docx]

**Supplementary file 5** List of WRKY33 target genes encoding transcription factors.

| Gene | Description | score_ChIP | log_2_FC Bc KO-WT |
| --- | --- | --- | --- |
| AT1G25560 | EDF1 | 24,29 | -0,42 |
| AT2G41710 | AP2-like ERF | 12,46 | -0,18 |
| AT4G37750 | ANT | 24,36 | -0,05 |
| AT5G07580 | ERF/AP2 | 11,97 | -0,98 |
| AT1G04370 | ATERF14 | 17,63 | -0,71 |
| AT4G17500 | ATERF-1 | 50,34 | 0,01 |
| AT3G23240 | ATERF1 | 35,69 | -0,13 |
| AT5G47230 | ATERF-5 | 21,67 | -1,35 |
| AT5G53290 | CRF3 | 28,46 | 0,36 |
| AT5G25190 | ESE3 | 16,85 | -0,97 |
| AT1G71520 | ERF020 | 16,21 | 0,22 |
| AT1G33760 | ERF022 | 19,47 | -0,95 |
| AT1G64380 | ERF061 | 12,53 | 1,14 |
| AT1G06160 | ORA59 | 21,31 | -2,45 |
| AT3G23220 | ESE1 | 12,60 | 0,15 |
| AT5G43410 | ERF096 | 14,94 | -1,14 |
| AT4G34410 | RRTF1 | 18,27 | 1,16 |
| AT2G33710 | ERF112 | 29,24 | -0,37 |
| AT5G07310 | ERF115 | 31,44 | n.d. |
| AT1G68550 | CRF10 | 17,35 | -0,75 |
| AT1G78080 | RAP2.4 | 21,24 | 0,44 |
| AT3G27785 | MYB118 | 12,46 | n.d. |
| AT3G23250 | ATMYB15 | 24,71 | 1,28 |
| AT1G66230 | AtMYB20 | 22,37 | -0,44 |
| AT1G74650 | ATMYB31 | 19,12 | -0,74 |
| AT5G06100 | ATMYB33 | 12,89 | 0,03 |
| AT4G12350 | AtMYB42 | 19,61 | -0,56 |
| AT5G16600 | AtMYB43 | 11,05 | 0,29 |
| AT1G18570 | AtMYB51 | 37,46 | -0,50 |
| AT1G68320 | AtMYB62 | 32,85 | 0,09 |
| AT5G11050 | MYB64 | 23,86 | n.d. |
| AT5G65790 | ATMYB68 | 17,28 | -2,64 |
| AT2G23290 | AtMYB70 | 13,45 | -0,84 |
| AT4G37260 | ATMYB73 | 17,21 | 0,62 |
| AT4G05100 | AtMYB74 | 14,94 | 0,85 |
| AT4G22680 | AtMYB85 | 24,99 | -0,69 |
| AT5G62470 | ATMYB96 | 13,31 | 0,34 |
| AT5G05090 | MYB | 17,91 | 1,39 |
| AT3G10760 | MYB | 25,28 | -0,21 |
| AT1G25550 | MYB like | 15,44 | 0,62 |
| AT4G38620 | ATMYB4 | 18,34 | 0,38 |
| AT1G14350 | AtMYB124 | 28,11 | 0,70 |
| AT1G14600 | MYB like | 19,61 | -0,49 |
| AT5G24110 | ATWRKY30 | 29,88 | 0,01 |
| AT2G04880 | ATWRKY1 | 16,78 | 0,26 |
| AT4G01250 | AtWRKY22 | 23,51 | 0,47 |
| AT2G40740 | ATWRKY55 | 12,53 | 1,88 |
| AT4G31550 | ATWRKY11 | 22,87 | 0,50 |
| AT2G23320 | AtWRKY15 | 33,14 | 0,16 |
| AT4G23550 | ATWRKY29 | 18,98 | 0,38 |
| AT2G38470 | ATWRKY33 | 30,45 | -3,32 |
| AT5G22570 | ATWRKY38 | 13,52 | 4,50 |
| AT4G11070 | AtWRKY41 | 35,61 | 2,72 |
| AT4G04450 | AtWRKY42 | 11,68 | 0,09 |
| AT5G49520 | ATWRKY48 | 19,26 | 1,38 |
| AT5G26170 | ATWRKY50 | 23,65 | 3,56 |
| AT4G23810 | ATWRKY53 | 49,21 | 1,45 |
| AT1G29280 | ATWRKY65 | 26,62 | -0,04 |
| AT1G80590 | ATWRKY66 | 14,51 | n.d. |
| AT1G29860 | ATWRKY71 | 17,63 | 0,49 |
| AT5G13080 | ATWRKY75 | 30,02 | -0,38 |
| AT3G15510 | ANAC056 | 18,55 | -0,93 |
| AT1G61110 | ANAC25 | 35,83 | n.d. |
| AT5G13180 | ANAC083 | 22,09 | -0,20 |
| AT2G43000 | ANAC042 | 15,15 | -0,51 |
| AT3G15500 | ANAC055 | 24,71 | -0,96 |
| AT5G22380 | NAC090 | 12,82 | 3,49 |
| AT3G44350 | NAC061 | 21,81 | 3,89 |
| AT3G12910 | NAC | 17,63 | -0,12 |
| AT1G35560 | TCP23 | 25,49 | -0,50 |
| AT3G15030 | MEE35 | 35,83 | -0,28 |
| AT5G23280 | TCP7 | 13,31 | -0,89 |
| AT2G45680 | TCP9 | 17,42 | -0,19 |
| AT1G61660 | bHLH112 | 19,61 | 0,97 |
| AT2G43140 | bHLH129 | 21,60 | 1,21 |
| AT2G40200 | bHLH51 | 18,83 | -0,23 |
| AT4G29100 | bHLH68 | 11,97 | -0,28 |
| AT5G56960 | bHLH041 | 36,96 | 0,59 |
| AT2G31730 | bHLH | 21,81 | 0,23 |
| AT3G20770 | AtEIN3 | 44,11 | 0,36 |
| AT4G38900 | bZIP like | 20,04 | 0,26 |
| AT1G42990 | ATBZIP60 | 50,63 | 0,47 |
| AT5G28770 | AtbZIP63 | 31,01 | -1,53 |
| AT1G78600 | BBX22 | 16,00 | -0,62 |
| AT3G19580 | AZF2 | 16,64 | 1,38 |
| AT5G66730 | ENY | 12,53 | 0,27 |
| AT3G60580 | C2H2-like | 19,75 | 0,42 |
| AT2G01940 | ATIDD15 | 17,13 | -1,20 |
| AT5G60470 | C2H2 and C2HC | 12,82 | 0,64 |
| AT5G04340 | C2H2 | 15,22 | 0,04 |
| AT2G40140 | ATSZF2 | 19,90 | 0,76 |
| AT3G55980 | ATSZF1 | 42,77 | 0,16 |
| AT4G29190 | AtC3H49 | 18,48 | -0,99 |
| AT5G46910 | C5HC2 type | 17,13 | 0,45 |
| AT1G30810 | JMJ18 | 18,62 | 0,52 |
| AT1G51700 | ADOF1 | 42,06 | 0,80 |
| AT4G24060 | DOF4.6 | 16,78 | 0,50 |
| AT5G02460 | DOF5.1 | 11,97 | -0,15 |
| AT4G27310 | BBX28 | 13,24 | -0,61 |
| AT4G39070 | BBX20 | 13,24 | 0,75 |
| AT2G41310 | ARR8 | 28,18 | -0,12 |
| AT5G24470 | APRR5 | 12,25 | 0,30 |
| AT1G21450 | SCL1 | 25,77 | 0,61 |
| AT5G59450 | SCL11 | 26,98 | -0,23 |
| AT1G07530 | ATGRAS2 | 13,03 | -0,36 |
| AT3G46600 | SCL30 | 16,43 | 0,31 |
| AT5G48150 | PAT1 | 26,55 | -0,02 |
| AT4G17230 | SCL13 | 33,49 | 0,35 |
| AT1G07520 | GRAS | 13,38 | 0,27 |
| AT3G06740 | GATA15 | 15,65 | -0,13 |
| AT4G17570 | GATA26 | 17,63 | 0,29 |
| AT5G66320 | GATA5 | 24,99 | -0,06 |
| AT3G54810 | BME3 | 16,92 | -0,04 |
| AT5G39760 | AtHB23 | 20,67 | -0,14 |
| AT4G00730 | AHDP | 13,45 | -0,24 |
| AT3G61890 | ATHB-12 | 13,24 | 0,08 |
| AT4G37790 | HAT22 | 14,23 | -0,13 |
| AT3G60390 | HAT3 | 15,79 | 0,77 |
| AT5G44180 | RLT2 | 11,12 | 0,31 |
| AT3G18010 | WOX1 | 32,50 | -0,44 |
| AT2G43500 | RWP-RK | 23,22 | -0,09 |
| AT1G76350 | RWP-RK | 16,71 | 0,01 |
| AT2G36960 | TKI1 | 29,81 | 0,29 |
| AT5G46760 | MYC3 | 16,78 | -0,31 |
| AT2G22770 | NAI1 | 12,53 | 0,64 |
| AT2G27100 | SE | 17,21 | -0,01 |
| AT4G18880 | AT-HSFA4A | 78,59 | 0,76 |
| AT1G06040 | BBX24 | 15,44 | -0,34 |
| AT3G14020 | NF-YA6 | 16,14 | -0,60 |
| AT1G55110 | AtIDD7 | 10,13 | -0,17 |
| AT4G23800 | 3xHMG-box2 | 16,78 | 1,25 |
| AT1G80420 | ATXRCC1 | 45,88 | -0,28 |
| AT2G22630 | AGL17 | 13,03 | 1,69 |

Note: Transcription factors highlighted in red color indicate WRKY33-regulated direct target genes.
