# Supplementary material for: Negative regulation of ABA signaling by WRKY33 is critical for Arabidopsis immunity towards Botrytis cinerea 2100
Source: eLife. 2015 Jun 15;4:e07295. doi: 10.7554/eLife.07295 (PMC4487144; doi:10.7554/eLife.07295)
Supplement: Supplementary file 6. — List of primers used for genotyping. DOI: http://dx.doi.org/10.7554/eLife.07295.028 [file elife07295s006.docx]

| Gene | Locus | Forward primer (5' - 3') | Reverse primer (5' - 3') |
| --- | --- | --- | --- |
| gh3.2 | AT4G37390 | TACGTAACCACCGGAACTTTG | AGAGCGGATGATTGTTGATTG |
| gh3.3 | AT2G23170 | TTTTAACGTATTAATCTTGGCACG | GGGAACAACAACATGATCCCT |
| nced3 | AT3G14440 | ACAGAGGCTCTCCTCCGTAAC | GTCAGCCACGAGAAGCTACAC |
| nced5 | AT1G30100 | TAACACCAAACCCAACCAAAC | TGACTCAACCCAAACCATCTC |
| wrky33 | AT2G38470 | CTCCTTCTCTTGTCTCTCCTTCC | TTGTGATTAAAGCTCCTGTGGTT |
| SALKLBb1.3 |  | ATTTTGCCGATTTCGGAAC |  |
| GK-LB8409 |  | ATATTGACCATCATACTCATTGC |  |

**Supplementary file 6** List of primers used for genotyping.
