# Supplementary material for: Negative regulation of ABA signaling by WRKY33 is critical for Arabidopsis immunity towards Botrytis cinerea 2100
Source: eLife. 2015 Jun 15;4:e07295. doi: 10.7554/eLife.07295 (PMC4487144; doi:10.7554/eLife.07295)
Supplement: Supplementary file 7. — List of primers used for ChIP-qPCR. DOI: http://dx.doi.org/10.7554/eLife.07295.029 [file elife07295s007.docx]

**Supplementary file 7** List of primers used for ChIP-qPCR.

| Gene | Locus | Forward primer (5’ – 3’) | Reverse primer (5’ – 3’) |
| --- | --- | --- | --- |
| CYP71A12 | AT2G30750 | caagtttgactgacccatct | gatttagaagtttgaataaccc |
| CYP71A13 | AT2G30770 | Ggtcgcattcaacagctaag | Ggaagtttgacttacccattca |
| PAD3 | AT3G26830 | AGGAGGTGTGCAATATGGAC | TAGGTTGCGCTGACCAAACA |
| AMT1 | AT5G05730 | TTAACATTCCGCTATCAGGCA | CGAACGGTCGTCTTTCTCAA |
| WRKY38 | AT5G22570 | CAAAAGTATATATTTGACTAAAGCTGG | TTAAACCAAGTAGTTGGAACGTAG |
| WRKY50 | AT5G26170 | GAATGGTACATAACAAGTCCTC | TGTGTTTCCAAGCAATAGAGACAT |
| WRKY53 P1 | AT4G23810 | AGTTAGGCTATTTAATGCGTATATCT | CATTGCTTTCAATCCCTTTGATAT |
| WRKY53 P2 | AT4G23810 | GAGAGTGACGCCATTATAAAAATTA | CAAAAAGAAAATCAATATTCAAAAGGAC |
| WRKY53 P3 | AT4G23810 | cctttgaccttatactctttcacta | TTGACCAAATGACCAAACCATAAAT |
| WRKY41 | AT4G11070 | GGTTAAACTAAAATAAACCCAAAGTTG | TGAAAATTTGACCAAGTGAGCAAAC |
| WRKY48 | AT5G49520 | GCTTGGTTGAATAACTGATGGT | ATGATTGACCACAGGATCATAG |
| WRKY55 | AT2G40740 | GTAATGTTGAAATTTGAAAGAAATAA  ATAATC | TCTAACTAATGATAACCCATTGACC |
| NAC061 | AT3G44350 | AAAGCACTCACTGTCTCACGTAT | GGCCCATCTTTGTGATAATTTC |
| NAC090 | AT5G22380 | AACTGGCAAGCCAAATTAAAAGAT | CGGATATTGGTAAAGACAAGGA |
| AKT1 | AT2G26650 | AAATGTTGGTTTCCACGTTT | GGAGCTTTAGTCGTCAAGTAGTT |
| NCED3 P1 | AT3G14440 | CCATGCTTTAGTGACGTTTACTTG | TTACTTCCGATAAACAGCTTCAATC |
| NCED3 P2 | AT3G14440 | TTGTCGGGTTGGTGTCCTC | AAATACAGTTGCCGGTCAAAGA |
| NCED5 | AT1G30100 | CCGGTAAATATTGTGAACCTTT | CCAAGTAACTGTCACCAATCAC |
| CYP707A3 P1 | AT5G45340 | TTTGTCGATCTCTGACCGATTT | TCACTTTAACGAAGCGCAAAC |
| CYP707A3 P2 | AT5G45340 | TCACAGGCAGAAAGTCAATTT | CCTATATATGTGCTTAGAACTTAATCAGA |
| WAKL7 | AT1G16090 | AGTTGACAAAGTAAGAGGCAGGA | ACAGTTGCCTACTTGGACCAC |
| RING1A | AT5G44280 | AAATTCAACCATTCACCGTTT | ACAATTCAAACCGGCAGC |
| NPR3 | AT5G45110 | TCCTTGTCCAAAGTTTCTTCC | TCAACCAAACCCAGAACCA |
| AAO1 | AT5G20960 | TCTTACCTCCTCGGATTCAAT | ACTTGTATTTCTTCTAGTCGTCCAC |
| ACS2 | AT1G01480 | TGCAGACTACAACATACCAAATG | CCAAGGCTATTTCTAAGCAACTT |
| JRG21 | AT3G55970 | CAACGACTAAGACGCTCTCACA | GCAAAGAATGTAATCATAGACGTGC |
| ERF5 | AT5G47230 | AAACATGAGATATTGACCGGC | TCGACTTGTTCAGACAGAATCAC |
| ERF1 | AT3G23240 | AGGATTGTCTTTAAGCATGTGC | CGCTCCTCAATACTCATGGA |
| ACS6 | AT4G11280 | TTCTTTCATGGAACTTTCGTTG | GATGTGTTGGGAAGTGAGATTG |
| LOX4 | AT1G72520 | ACGTTGAACATTAAAGTGTCCG | GCCTTGAGAAAGAGAGAGCA |
| WRKY33 | AT2G38470 | Aagcattgagccgcccctca | Atgaagaagagtagtttctgag |
| ORA59 | AT1G06160 | TGTGGACACCAAATGATAAAGAG | CAAGATTAAGAAGTTGAATTGGCTG |
| Reference | AT2G04450 | GAAGAGATTGCAGGAGATGG | TTGAGGAGGTTGGCGTGATC |
