# Supplementary material for: Negative regulation of ABA signaling by WRKY33 is critical for Arabidopsis immunity towards Botrytis cinerea 2100
Source: eLife. 2015 Jun 15;4:e07295. doi: 10.7554/eLife.07295 (PMC4487144; doi:10.7554/eLife.07295)
Supplement: Supplementary file 8. — List of primers used for qRT-PCR. DOI: http://dx.doi.org/10.7554/eLife.07295.030 [file elife07295s008.docx]

**Supplementary file 8** List of primers used for qRT-PCR.

| Gene | Locus | Forward primer (5’ – 3’) | Reverse primer (5’ – 3’) |
| --- | --- | --- | --- |
| WRKY38 | AT5G22570 | CATAACTTGAAAGCGGTCCAC | AAATGAACTCCCCACACGAA |
| WRKY50 | AT5G26170 | GATCTTGTGTCTGCGGTTTC | CAGAAGCAGTGGCTGTAGCA |
| WRKY53 | AT4G23810 | CCAGAGTCAAACCAGCCATTA | CGTATCAGGGAACGAGAAAAC |
| WRKY41 | AT4G11070 | CCGTCGGATTTCACTGGA | GCCTGTGTTAATCTCAGCCG |
| WRKY48 | AT5G49520 | TCAACATCACCAGCCCTACA | CATATCATAACCAAAGCCGGG |
| WRKY55 | AT2G40740 | CTTCCGGGTCACATACCGT | TGAAATCCATGTTGGTTCCG |
| NAC061 | AT3G44350 | CAGTATCTGTCGAATCTACA | TTGGTTTCCTCCTGAATGTG |
| NAC090 | AT5G22380 | GCTGAGACATCGTTCCGTG | CAGTCCCACATCGGTTCTG |
| GH3.2 | AT4G37390 | TGGAGCAGCAGAAGCATCAT | TGTCGCCAACTCTGTAACGG |
| GH3.3 | AT2G23170 | ACCTATGCTGGGCTTAACCGT | AGAGCGATGCGTTCTCAACC |
| NCED3 | AT3G14440 | GGAGAAGGAGGAGAGGAAGA | CGACCTGCTTCGCCAAATCAT |
| NCED5 | AT1G30100 | CGAGGAGAGTTGGGAATCGG | TGGTTTAACATATCCGCCGAA |
| CYP707A3 | AT5G45340 | CAGATGGTCAATCGTAGGGC | TTTCGTTCCAAGGCAATAGG |
| NPR3 | AT5G45110 | CGACATCCTCGACGATTTCC | CATGTTGTGTTGTGCAGGTCA |
| ACS2 | AT1G01480 | AGTTTCCGACGACTTTACGAG | GAAGAGGTGAGTGTGGTGACA |
| ERF1 | AT3G23240 | TCGGCGATTCTCAATTTTTCG | CCGTCTCATCGAGTGTTTCCT |
| ACS6 | AT4G11280 | AAACCGAACTATGGCGTGTG | TCATGGCAATGGAACGAAC |
| WRKY33 | AT2G38470 | CTCGTGGTAGCGGTTACGCC | CCTTTGCTCTAGAGAATCCACC |
| ICS1 | AT1G74710 | cattgatctatgcggggacag | tggacaaaagctcgtacctgag |
| NPR1 | AT1G64280 | AGGCACTTGACTCGGATGATATTG | CTTCACATTGCAATATGCAACAGC |
| NPR4 | AT4G19660 | TCGTATCCCGAGAAAGGCAC | AGCTGGTGATGAAGAAGAAAGACAA |
| TRXH5 | AT1G45145 | TGAATTGCAAGCTGTTGCTC | GCAGAAGCTACAAGACCACC |
| FMO1 | AT1G19250 | CGTCCAAAGCAGCTCGAAC | CGTGGAAATGCAATGACGTTT |
| PR2 | AT3G57260 | TTCAAACCCGTACGACACTG | tctggcgtcgtgaggaggaa |
| EDS1 | AT3G48090 | AAGCATGATCCGCACTCG | CGAAGACACAGGGCCGTA |
| PAD4 | AT3G52430 | GGTTCTGTTCGTCTGATGTTT | GTTCCTCGGTGTTTTGAGTT |
| NIMIN-1 | AT1G02450 | TTCAAACCCGTACGACACTG | tctggcgtcgtgaggaggaa |
| PR1 | AT2G14610 | TTCTTCCCTCGAAAGCTCAA | AAGGCCCACCAGAGTGTATG |
| NAC019 | AT1G52890 | GGAGGAAGTCGAGAGCAGTCA | CAAACCCACCAACTTGCCC |
| NAC055 | AT3G15500 | CGCAGCAACAAACTGAGGG | CCCGAGTACCCAAATCCGTT |
| MPK11 | AT1G01560 | CGATGAAGCCTTGTGCCA | CCTTGATGTTCTCTTCCGTCA |
| BIR1 | AT5G48380 | GGGTAACCTCGTGGAATGG | CTTCAAACATGGTTGGCCTC |
| CDPK1 | AT1G18890 | GGATGTCGATGGAAATGGG | CGCTTAGAACACTGGCGTCT |
| CRK36 | AT4G04490 | GACAAGGTGGGTTTGGATCTG | GTCCTGAGCCTCCAGCTAATC |
| ORA59 | AT1G06160 | AGGCAGCCTCGCAGTACTCAA | ctcttcaaggctatcaccgga |
| PDF1.2 | AT5G44420 | TGCATGATCCATGTTTGGCTC | acgcaccggcaatggtggaa |
| ABI1 | AT4G26080 | AAGGGAAAGATCCTGCGGC | TCCGAGGCTTCAAATCAACC |
| AMT1 | AT5G05730 | AGTGACCCGCAAGACGAA | TCACAAATGCAGATTCAGCC |
| EXP | AT4G26410 | GAGCTGAAGTGGCTTCCATGAC | GGTCCGACATACCCATGATCC |
